# Nomogram for Predicting COVID-19 Disease Progression Based on Single-Center Data: Observational Study and Model Development

**DOI:** 10.2196/19588

**Published:** 2020-09-08

**Authors:** Tao Fan, Bo Hao, Shuo Yang, Bo Shen, Zhixin Huang, Zilong Lu, Rui Xiong, Xiaokang Shen, Wenyang Jiang, Lin Zhang, Donghang Li, Ruyuan He, Heng Meng, Weichen Lin, Haojie Feng, Qing Geng

**Affiliations:** 1 Renmin Hospital Wuhan University Wuhan China

**Keywords:** coronavirus disease 2019, COVID-19, risk factors, nomogram

## Abstract

**Background:**

In late December 2019, a pneumonia caused by SARS-CoV-2 was first reported in Wuhan and spread worldwide rapidly. Currently, no specific medicine is available to treat infection with COVID-19.

**Objective:**

The aims of this study were to summarize the epidemiological and clinical characteristics of 175 patients with SARS-CoV-2 infection who were hospitalized in Renmin Hospital of Wuhan University from January 1 to January 31, 2020, and to establish a tool to identify potential critical patients with COVID-19 and help clinical physicians prevent progression of this disease.

**Methods:**

In this retrospective study, clinical characteristics of 175 confirmed COVID-19 cases were collected and analyzed. Univariate analysis and least absolute shrinkage and selection operator (LASSO) regression were used to select variables. Multivariate analysis was applied to identify independent risk factors in COVID-19 progression. We established a nomogram to evaluate the probability of progression of the condition of a patient with COVID-19 to severe within three weeks of disease onset. The nomogram was verified using calibration curves and receiver operating characteristic curves.

**Results:**

A total of 18 variables were considered to be risk factors after the univariate regression analysis of the laboratory parameters (*P*<.05), and LASSO regression analysis screened out 10 risk factors for further study. The six independent risk factors revealed by multivariate Cox regression were age (OR 1.035, 95% CI 1.017-1.054; *P*<.001), CK level (OR 1.002, 95% CI 1.0003-1.0039; *P*=.02), CD4 count (OR 0.995, 95% CI 0.992-0.998; *P*=.002), CD8 % (OR 1.007, 95% CI 1.004-1.012, *P*<.001), CD8 count (OR 0.881, 95% CI 0.835-0.931; *P*<.001), and C3 count (OR 6.93, 95% CI 1.945-24.691; *P*=.003). The areas under the curve of the prediction model for 0.5-week, 1-week, 2-week and 3-week nonsevere probability were 0.721, 0.742, 0.87, and 0.832, respectively. The calibration curves showed that the model had good prediction ability within three weeks of disease onset.

**Conclusions:**

This study presents a predictive nomogram of critical patients with COVID-19 based on LASSO and Cox regression analysis. Clinical use of the nomogram may enable timely detection of potential critical patients with COVID-19 and instruct clinicians to administer early intervention to these patients to prevent the disease from worsening.

## Introduction

### Background

COVID-19 is a respiratory illness that is caused by the novel virus SARS-CoV-2; it was first reported in December 2019 in Wuhan, Hubei Province, China [1-5]. Although governments of countries worldwide have called for and taken relevant measures to stop the spread of the disease, the epidemic has not been effectively controlled [6-8]. Symptoms of COVID-19 range from mild cough to pneumonia; patients may even be asymptomatic [3,9]. There is evidence that this disease can be spread from person to person [10]. Whole genome sequencing showed that SARS-CoV-2 is a beta coronavirus that is similar to human severe acute respiratory syndrome coronavirus (SARS-CoV) and Middle East respiratory syndrome coronavirus (MERS-CoV). This new coronavirus evolved from SARS-CoV and MERS-CoV and requires enhanced surveillance and further investigation [11]. Like several other coronaviruses, SARS-CoV-2 initially causes mild or moderate symptoms in most patients [12,13]. To date, only a small percentage of patients with SARS-CoV-2 infection have developed severe pneumonia. Although the average incubation period of SARS-CoV-2 in the human body is 14 days, some patients progress rapidly to respiratory failure once they become infected. Recognition of the risk factors that promote COVID-19 progression and early intervention of this disease may prevent its exacerbation. Understanding these factors is also very important to reduce the proportion of critically ill patients and to improve the cure rate.

### Study Goals

The aim of this study was to summarize the epidemiological and clinical characteristics of 175 patients with SARS-CoV-2 infection who were hospitalized in Renmin Hospital of Wuhan University from January 1 to January 31, 2020. The study also aimed to explore the independent risk factors in COVID-19 progression and accurately assess the incidence of severe SARS-CoV-2 infection. In addition, a new risk-predictive model was established to screen out potential critical patients for early intervention.

## Methods

### Patient Recruitment

**Figure 1:**
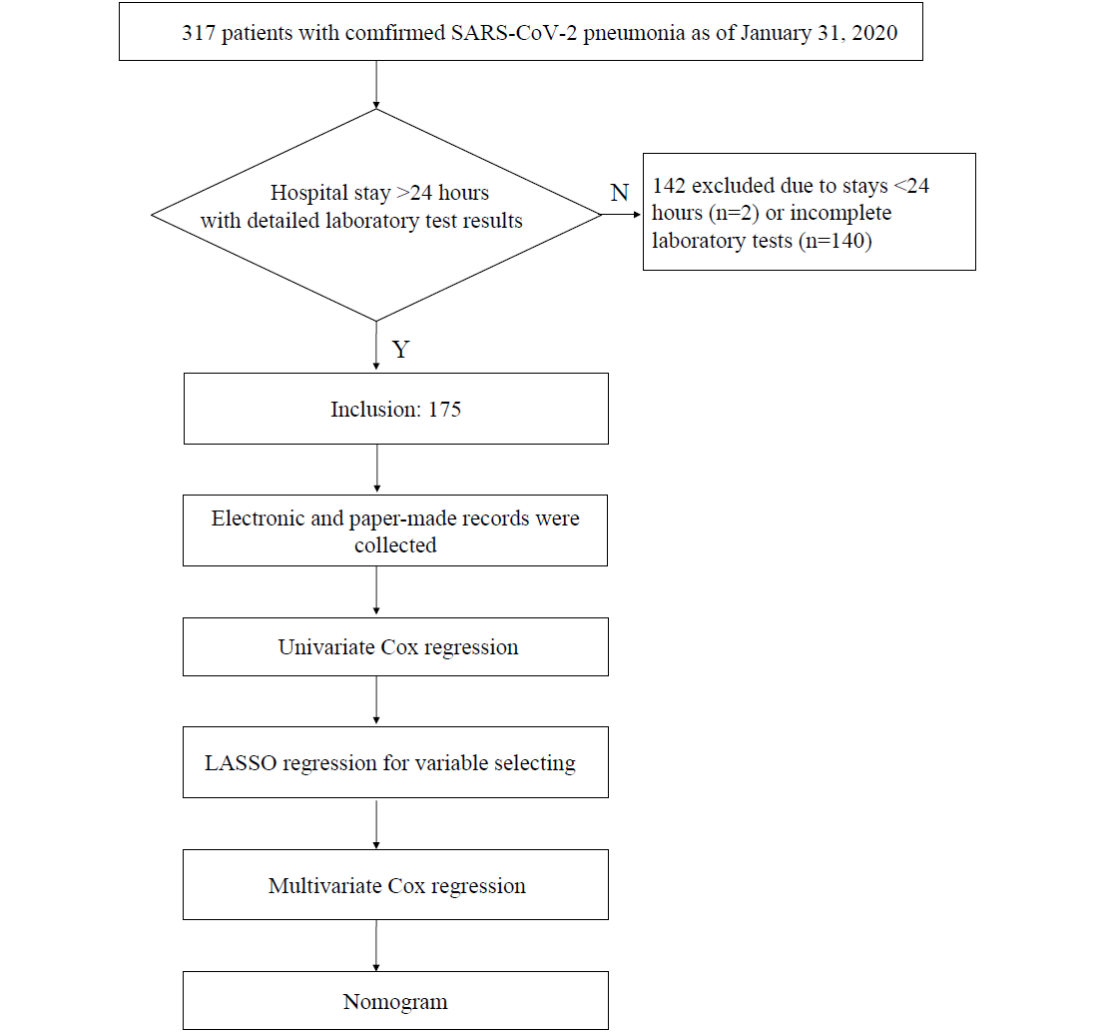
Study flowchart. LASSO: least absolute shrinkage and selection operator.

For this retrospective single-center study, 175 patients who were hospitalized from January 1 to January 31, 2020, in Renmin Hospital of Wuhan University were enrolled. Patients who were hospitalized for less than 24 hours or who lacked detailed laboratory test results were excluded (Figure 1). All the patients in this study were diagnosed according to the World Health Organization (WHO) interim guidance [14]. All the patients with COVID-19 were diagnosed using a reverse transcriptase–polymerase chain reaction (RT-PCR) assay for SARS-CoV-2 according to the Pneumonitis Diagnosis and Treatment Plan for SARS-CoV-2 Infection (Trial Version 5) issued by the National Health Commission of the People’s Republic of China (NHCPRC). The test was performed using specific steps described previously [9]. All the clinical features, radiological characteristics, clinical laboratory results, and outcome data of the 175 patients were obtained from electronic medical records. Detailed patient information was collected, including past medical history, current medical history, laboratory findings, imaging data, treatment measures, symptoms, signs, and immune function test results. Follow-up was initiated from suspicion of infection or confirmed diagnosis to the time when the patient’s condition became severe to the time of discharge or to January 31, 2020. We recorded the baselines of these tests, with the first value being within three days of onset admission.

### Statistical Analysis

Frequency rates were used to describe categorical variables, and means were used to describe continuous variables. Differences between groups were tested using the chi-square test, *t* test, or Mann-Whitney U test. Univariate analysis, least absolute shrinkage and selection operator (LASSO) regression, and multivariate Cox regression were used to screen for independent risk factors. The statistical analyses were performed using GraphPad Prism 8 (GraphPad Software). Nomogram, calibration, and receiver operating characteristic (ROC) curves were established using R version 3.6.1 (R Project). The risk score was calculated using multivariate Cox regression. The cutoff values of independent risk factors were based on the maximum Youden index. A 2-sided α <.05 was considered to indicate statistical significance.

## Results

### Patient Characteristics

All 175 patients in this study were confirmed to be infected with SARS-CoV-2. The average age of the 175 patients was 46.9 years (SD 17.33). Severe or critical patients were significantly older than mild or moderate patients (*P*<.001). Critical patients had a wider range of lung lesions than milder patients (*P*<.001). Chest tightness was more common in severe or critical patients than in mild or moderate patients (*P*=.001). Table 1 shows comparisons of the significant characteristics between the two groups of patients enrolled in the study (*P*<.05).

**Table 1:** Baseline characteristics of the patients infected with SARS-CoV-2 (N=175).

Participant characteristics		Values by disease severity				*P* value
		Mild (n=27)	Moderate (n=72)	Severe (n=18)	Critical (n=58)	
**Sex, n (%)**						.64
	Female	13 (48.1)	41 (56.9)	11 (61.1)	28 (48.3)	
	Male	14 (51.9)	31 (43.1)	7 (38.9)	30 (51.7)	
Age (years), median (IQR)		31 (29-42)	35 (30-50.75)	51 (37.75-61)	62 (47-71)	<.001
**Age (years), n (%)**						<.001
	≤30	11 (40.7)	21 (29.2)	1 (5.6)	3 (5.2)	
	31-40	8 (29.6)	25 (34.7)	4 (22.2)	7 (12.1)	
	41-50	4 (14.8)	8 (11.1)	4 (22.2)	6 (10.3)	
	51-60	1 (3.7)	13 (18.1)	5 (27.8)	10 (17.2)	
	>60	3 (11.1)	5 (6.9)	4 (22.2)	32 (55.2)	
**Computed tomography scan, n (%)**						<.001
	Normal	27 (100)	0 (0)	0 (0)	0 (0)	
	One lobe infected	0 (0)	25 (34.7)	6 (33.3)	5 (8.6)	
	More than one lobe infected	0 (0)	47 (65.3)	12 (66.7)	53 (91.4)	
**Fever, n (%)**						.01
	Yes	17 (63)	43 (59.7)	14 (77.8)	49 (84.5)	
	No	10 (37)	29 (40.3)	4 (22.2)	9 (15.5)	
**Dry cough, n (%)**						.07
	Yes	2 (7.4)	11 (15.3)	3 (16.7)	17 (29.3)	
	No	25 (92.6)	61 (84.7)	15 (83.3)	41 (70.7)	
**Expectoration, n (%)**						.007
	Yes	5 (18.5)	28 (38.9)	10 (55.6)	33 (56.9)	
	No	22 (81.5)	44 (61.1)	8 (44.4)	25 (43.1)	
**Pharyngalgia, n (%)**						.25
	Yes	4 (14.8)	11 (15.3)	0 (0)	5 (8.6)	
	No	23 (85.2)	61 (84.7)	18 (100)	53 (91.4)	
**Chest tightness, n (%)**						.001
	Yes	6 (22.2)	8 (11.1)	7 (38.9)	23 (39.7)	
	No	21 (77.8)	64 (88.9)	11 (61.1)	35 (60.4)	
**Myalgia, n (%)**						.80
	Yes	4 (14.8)	6 (8.3)	2 (11.1)	7 (12.1)	
	No	23 (85.2)	66 (91.7)	16 (88.9)	51 (87.9)	
**Fatigue, n (%)**						.62
	Yes	8 (29.6)	18 (25)	3 (16.7)	11 (19)	
	No	19 (70.4)	54 (75)	15 (83.3)	47 (81)	
**Diarrhea, n (%)**						.55
	Yes	3 (11.1)	5 (6.9)	0 (0)	4 (6.9)	
	No	24 (88.9)	67 (93.1)	18 (100)	54 (93.1)	
**Headache, n (%)**						.08
	Yes	6 (22.2)	11 (15.3)	1 (5.6)	3 (5.2)	
	No	21 (77.8)	61 (84.7)	17 (94.4)	55 (94.8)	
						

### Laboratory Parameters

The baseline laboratory tests are shown in Table 2. As the patients’ conditions worsened, their lymphocyte counts significantly decreased, while their C-reactive protein, lactate dehydrogenase, and creatine kinase (CK) levels increased significantly (Table 2). More importantly, the CD3 (count and ratio), CD4 (count and ratio), CD8 (count and ratio), and CD19 (count and ratio) values of severe or critical patients were lower than those of the mild or moderate patients (Table 2). In addition, severe or critical patients had higher IgG levels than mild or moderate patients (Table 2).

**Table 2:** Laboratory findings of patients infected with SARS-CoV-2 on admission to hospital (N=175).

Variable	Normal range	Values by disease severity, mean (95% CI)				*P* value
		Mild	Moderate	Severe	Critical	
White blood cell count, ×10^9^/L	3.5-9.5	5.16 (4.47 to 5.86)	4.73 (4.39 to 5.07)	5.37 (4.1 to 6.64)	5.22 (4.69 to 5.75)	.35
Neutrophil count, ×10^9^/L	1.8-6.3	2.98 (2.35 to 3.61)	2.62 (2.38 to 2.87)	3.95 (2.64 to 5.26)	3.83 (3.31 to 4.35)	<.001
Lymphocyte count, ×10^9^/L	1.1-3.2	1.55 (1.33 to 1.77)	1.58 (1.41 to 1.74)	1.02 (0.78 to 1.26)	0.96 (0.82 to 1.09)	<.001
Platelet count, ×10^9^/L	125-350	209.2 (182.7 to 235.7)	208.1 (194.4 to 221.7)	211.9 (169.9 to 253.9)	186.9 (171.0 to 202.7)	.19
C-reactive protein, mg/L	0-5	4.99 (1.02 to 8.95)	9.21 (5.87 to 12.55)	31.1 (13.98 to 48.22)	43.24 (32.70 to 53.77)	<.001
Alanine aminotransferase, U/L	9-50	24.93 (15.78 to 34.07)	22.15 (17.26 to 27.05)	29.67 (20.58 to 38.75)	28.64 (23.13 to 34.15)	.28
Aspartate aminotransferase, U/L	15-40	24.85 (20.06 to 29.64)	22.85 (20.40 to 25.30)	29.61 (22.93 to 36.29)	33.24 (28.66 to 37.82)	<.001
Urea, mmol/L	3.1-8.0	3.71 (3.11 to 4.3)	4.05 (3.79 to 4.3)	6.17 (4.6 to 7.74)	5.74 (4.36 to 7.12)	.003
Creatinine, μmol/L	57-97	52.41 (46.13 to 58.68)	54.44 (51.71 to 57.18)	68.06 (54.75 to 81.36)	94.14 (50.26 to 138.0)	.1
Lactate dehydrogenase, U/L	120-250	186.2 (165.1 to 207.2)	193.8 (182.4 to 205.2)	242.1 (201.1 to 283.0)	294.1 (262.7 to 325.5)	<.001
Creatine kinase, U/L	50-310	64.96 (24.26 to 105.7)	71.03 (58.94 to 83.11)	87.67 (45.81 to 129.5)	123.4 (83.10 to 163.7)	.03
CD3 (%)	56-86	72.15 (68.45 to 75.84)	70.8 (68.62 to 72.99)	67.68 (61.38 to 73.99)	58.19 (54.36 to 62.03)	<.001
CD3 count, /μL	723-2737	1124 (923.0 to 1326)	1036 (933.0 to 1140)	658.4 (465.9 to 851.0)	577.1 (460.1 to 694.1)	<.001
CD4 (%)	33-58	41.71 (38.81 to 44.61)	41.19 (39.18 to 43.20)	41.85 (37.44 to 46.26)	31.91 (29.23 to 34.60)	<.001
CD4 count, /μL	404-1612	669.9 (573.6 to 766.3)	610.8 (544.8 to 676.7)	402.7 (288.5 to 517.0)	315 (247.2 to 382.7)	<.001
CD8 (%)	13-39	27.06 (24.62 to 29.51)	25.75 (24.41 to 27.08)	22.64 (18.63 to 26.64)	23.98 (20.97 to 26.98)	.21
CD8 count, /μL	220-1129	444.3 (355.1 to 533.5)	369.8 (328.7 to 410.8)	227.6 (151.7 to 303.4)	237.9 (183.9 to 291.9)	<.001
CD4/CD8 ratio	0.9-2.0	1.64 (1.43 to 1.85)	1.93 (1.46 to 2.4)	2.55 (1.28 to 3.81)	1.63 (1.38 to 1.87)	.17
CD19 (%)	5-22	14.04 (11.53 to 16.55)	13.21 (12.00 to 14.43)	21.82 (15.30 to 28.34)	15.46 (13.39 to 17.54)	<.001
CD19 count, /μL	80-616	210 (169.4 to 250.5)	197.1 (163.0 to 231.2)	178.2 (120.0 to 236.4)	129 (108.4 to 149.6)	.004
CD16+56 (%)	5-26	17.66 (3.458 to 31.86)	13.09 (11.22 to 14.97)	8.92 (6.227 to 11.61)	23.85 (20.21 to 27.49)	<.001
CD16+56 count, /μL	84-724	161.3 (82.20 to 240.3)	183.5 (150.4 to 216.5)	82.78 (55.13 to 110.4)	190 (154.7 to 225.3)	.04
IgG, g/L	8-16	11.83 (10.32 to 13.34)	11.81 (11.01 to 12.62)	18.08 (15.44 to 20.73)	13.79 (12.64 to 14.93)	<.001
IgM, g/L	0.4-3.45	1.19 (1.03 to 1.35)	1.2 (1.07 to 1.33)	1.08 (0.74 to 1.41)	1.13 (1.0 to 1.27)	.79
IgA, g/L	0.76-3.9	1.9 (1.55 to 2.25)	4.49 (–0.42 to 9.39)	1.83 (1.44 to 2.22)	2.23 (1.94 to 2.52)	.71
IgE, IU/mL	<100	71.7 (27.92 to 115.5)	88.89 (41.23 to 136.6)	59.83 (8.444 to 111.2)	84.1 (54.48 to 113.7)	.89
Complement C3, g/L	0.81-1.6	0.78 (0.72 to 0.84)	0.83 (0.79 to 0.87)	0.89 (0.81 to 0.98)	0.88 (0.82 to 0.94)	.09
Complement C4, g/L	0.1-0.4	0.2 (0.17 to 0.23)	0.24 (0.22 to 0.27)	0.22 (0.19 to 0.25)	0.28 (0.25 to 0.31)	.003

### Screening for Independent Risk Factors and Constructing a Predictive Nomogram

**Figure 2:**
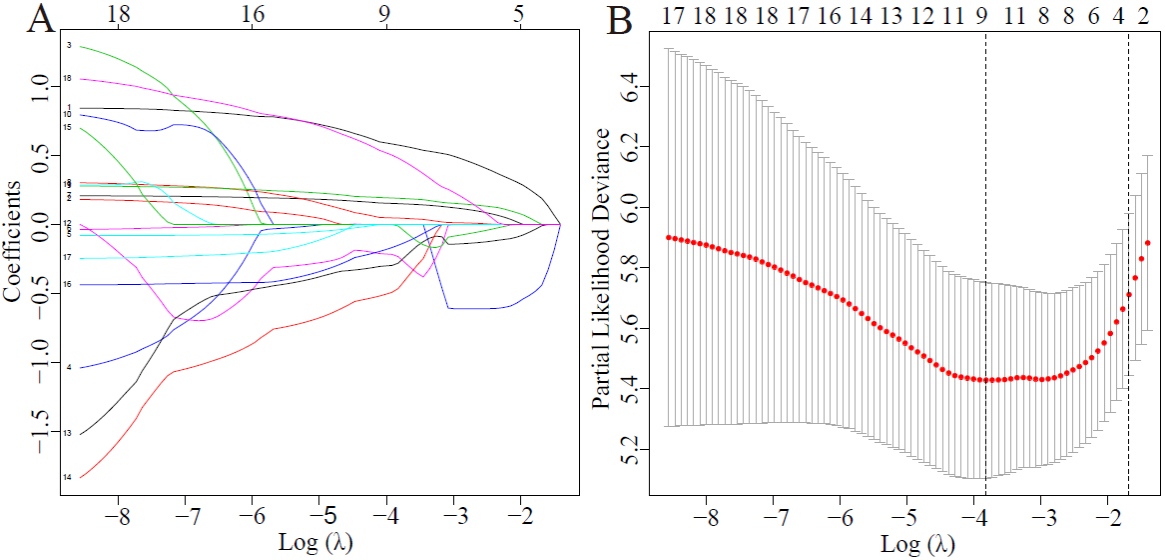
(A) The log (λ) values of the 18 parameters as shown using least absolute shrinkage and selection operator (LASSO) coefficient profiles. (B) The most suitable log (λ) values for variable selection based on the LASSO Cox regression.

The 175 patients were divided into a Mild group and a Severe group according to disease severity. Patients with mild and moderate illness were included in the Mild Group (nonsevere illness), and patients with severe and critical illness were included in the Severe Group. A total of 18 variables were considered to be risk factors as revealed by the univariate analysis (Multimedia Appendix 1). We performed LASSO Cox regression to further select variables (Figure 2), followed by multivariate Cox regression analysis. Multimedia Appendix 2 shows the results of the multivariate analysis. With older age (odds ratio [OR] 1.035, 95% CI 1.017-1.054); higher levels of blood CK (OR 1.002, 95% CI 1.0003-1.0039), CD8 % (OR 1.007, 95% CI 1.004-1.012), and C3 (OR 6.93, 95% CI 1.945-24.691); and lower levels of CD4 (OR 0.995, 95% CI 0.992-0.998) and CD8 (OR 0.881, 95% CI 0.835-0.931), a patient would be more likely to progress to severe disease within three weeks of disease onset.

**Figure 3:**
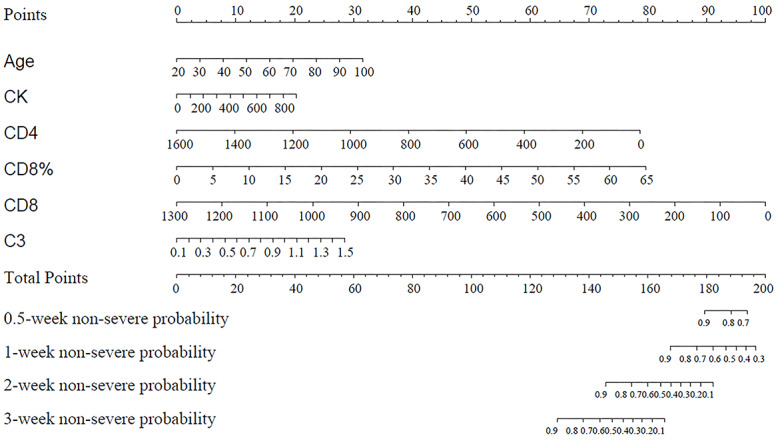
Establishment of the nomogram based on the six independent risk factors resulting from multivariate Cox regression to predict the 0.5-, 1-, 2-, and 3-week nonsevere probabilities for patients with COVID-19 in the developing set. Each selected variable is represented by a line in the figure. According to the value, each variable receives 1 point. The total points are added for each variable and matched with the probability of COVID-19 progression.

Figure 3 shows the nomogram of the multivariate Cox regression model. All independent risk factors have their own lines in the nomogram, with each receiving a point according to value. The total points are added and match the probability of COVID-19 progression. An example is shown in Multimedia Appendix 3, and the calibration curves are shown in Multimedia Appendix 4. The apparent value is close to the ideal value, which indicates good predictive performance of the model. Multimedia Appendix 5 shows the areas under the ROC curves of the nomogram. The curves proved that this model obtained in the study had good predictive performance for COVID-19 progression within three weeks.

## Discussion

### Principal Results

In this report, we found that age, CK level, CD4 count, CD8 count, CD8 %, and C3 count were the independent risk factors for the progression of COVID-19. In addition, we established a nomogram based on the six independent risk factors to predict the probability of 0.5-, 1-, 2-, and 3-week nonsevere probability.

Since the first case of unexplained pneumonia was reported in the city of Wuhan [15], the disease has spread rapidly worldwide [16,17]. COVID-19 was recognized by the WHO as an international emergency public health event [18]. Current epidemiological studies have shown that the most common symptom of patients with COVID-19 before and after consultation is fever [9,15,19]. In this study, fever was identified in 123/175 patients (70.3%) when they were hospitalized. A total of 76/175 participants in this study (43.4%) were severely or critically ill, which accounts for the much higher rate of severity than that reported by Guan et al [19]. This may be due to the insufficient number of hospital beds and the fact that patients with severe conditions were preferentially admitted. Although the fatality rate of SARS-CoV-2 appears to be lower than that of SARS-CoV or MERS-CoV, the outcomes of SARS-CoV-2 patients are worse once the disease enters the severe stage. A retrospective study showed a 61.5% mortality rate in patients with severe COVID-19 [20]. If patients with high risk factors to progress to severe or critical illness can be screened out in a timely fashion for early intervention, the proportion of severe or critically ill patients and their mortality may be reduced significantly.

In this study, 175 patients were divided into a Mild group (patients with mild and moderate illness) and a Severe group (patients with severe and critical illness). A total of 33 variables were included in this study, including age and clinical laboratory parameters. Indices including age, CD4 count, CD8 count, CD8 %, C3 count, and CK level were filtered out using LASSO and multivariate Cox regression. The indices were considered to be independent risk factors that affect COVID-19 progression. It was reported in a study involving 1099 COVID-19 patients that severe patients were typically seven years older than nonsevere patients (median) and that older patients with COVID-19 were more likely to progress to severe illness [19]. Research has shown that T cells are reduced and eventually fail in COVID-19 patients [21]. The results of this study are similar to the two findings mentioned above.

Because no specific medicine or vaccine has been made available for the treatment of SARS-CoV-2 infection to date [22], it is necessary to predict independent risk factors for the early detection of potential patients with severe COVID-19 and provide early intervention. Based on this research, age, myocardial function, and immune system and complement system function are key factors that impact COVID-19 progression. This study presents a nomogram that may be helpful to clinical physicians. Early intervention and supportive treatment for patients whose age and CK, CD4, CD8 and C3 values are in high-risk ranges may have important significance in reducing the severity and mortality of COVID-19.

### Limitations

This study has some limitations. First, only 175 cases were included in the construction of the model that was used to screen for independent risk factors. Second, this is an observational study; therefore, it cannot directly lead to causal conclusions. Third, some patients had severe underlying diseases before becoming infected with the virus; therefore, there may be bias in the calculation of the nonsevere patients’ survival times.

### Conclusions

The COVID-19 outbreak quickly spread worldwide after it was first discovered in Wuhan. Currently, no specific medicine is available for the treatment of SARS-CoV-2 infection. It is necessary to establish a new predictive model that can be used to screen potential critical patients and provide early intervention. We presented a nomogram, compiled through the use of LASSO regression and multivariate Cox regression, which considers various clinical risk factors in evaluating the probability of COVID-19 progression. This nomogram may help clinical physicians prevent COVID-19 progression.

## Appendix

Multimedia Appendix 1 Univariate Cox regression for independent risk factors in COVID-19 progression.

Multimedia Appendix 2 Multivariate Cox regression for independent risk factors in COVID-19 progression.

Multimedia Appendix 3 Instructions for using the nomogram.

Multimedia Appendix 4 Calibration curves for evaluating the predictive performance of nomogram.

Multimedia Appendix 5 Receiver operating characteristic curves for evaluating the predictive performance of the nomogram.

## Abbreviations

CK: creatine kinase
LASSO: least absolute shrinkage and selection operator
MERS-CoV: Middle East respiratory syndrome coronavirus
NHCPRC: National Health Commission of the People’s Republic of China
OR: odds ratio
ROC: receiver operating characteristic
RT-PCR: reverse transcriptase–polymerase chain reaction
SARS-CoV: severe acute respiratory syndrome coronavirus
WHO: World Health Organization
