# Supplementary material for: Nomogram for Predicting COVID-19 Disease Progression Based on Single-Center Data: Observational Study and Model Development
Source: JMIR Med Inform. 2020 Sep 8;8(9):e19588. doi: 10.2196/19588 (PMC7485996; doi:10.2196/19588)
Supplement: Multimedia Appendix 1 [file medinform_v8i9e19588_app1.doc]

| **Table S1. Univariate Cox regression for independent risk factors in COVID-19 progression** | | |
| --- | --- | --- |
| **Variable** | **OR** | ***P* value** |
| CD3% | 0.9516 | <.001 |
| Creatine kinase (CK) | 1.0041 | <.001 |
| Age | 1.0344 | <.001 |
| CD16+56 % | 1.0220 | <.001 |
| Lactate dehydrogenase (LDH) | 1.0041 | <.001 |
| CD3 | 0.9985 | <.001 |
| CD4% | 0.9503 | <.001 |
| CD4 | 0.9977 | <.001 |
| C-reactive protein (CRP) | 1.0103 | <.001 |
| CD8 | 0.9973 | .001 |
| Lymphocyte | 0.4382 | .001 |
| Urea | 1.0777 | .002 |
| CD19% | 1.0389 | .009 |
| CD8% | 0.9662 | .01 |
| Neutrophil/Lymphocyte | 1.0399 | .01 |
| Leukocyte/Lymphocyte | 1.0379 | .02 |
| C3 | 3.6074 | .02 |
| Creatinine (Cr) | 1.0003 | .03 |
| Aspartate aminotransferase (AST) | 1.0093 | .06 |
| CD19 | 0.9977 | .08 |
| Ig G | 1.0362 | .12 |
| Neutrophil | 1.0815 | .15 |
| Alanine aminotransferase (ALT) | 1.0065 | .21 |
| CD4/CD8 | 1.1027 | .22 |
| Urea/Cr | 0.0213 | .23 |
| Ig E | 1.0007 | .27 |
| Ig M | 0.7440 | .27 |
| C4 | 2.2057 | .32 |
| Ig A | 0.9792 | .66 |
| CD16+56 | 1.0004 | .70 |
| ALT/AST | 0.9207 | .74 |
| Leukocyte | 1.0108 | .85 |
| Platelets | 0.9998 | .92 |
