# Supplementary material for: Nomogram for Predicting COVID-19 Disease Progression Based on Single-Center Data: Observational Study and Model Development
Source: JMIR Med Inform. 2020 Sep 8;8(9):e19588. doi: 10.2196/19588 (PMC7485996; doi:10.2196/19588)
Supplement: Multimedia Appendix 2 [file medinform_v8i9e19588_app2.doc]

| **Table S2. Multivariate Cox regression for independent risk factors in COVID-19 progression** | | | | | | | |
| --- | --- | --- | --- | --- | --- | --- | --- |
| **Variable** | | **OR** | | **Lower 95%** | **Upper 95%** | ***P*** |  |
| Age | | 1.0353 | | 1.0166 | 1.0543 | <.001 | *** |
| Creatinine (Cr) | | 1.0002 | | 0.9999 | 1.0004 | .32 |  |
| Lactate dehydrogenase (LDH) | | 1.0007 | | 0.9982 | 1.0033 | .57 |  |
| Creatine kinase (CK) | | 1.0021 | | 1.0003 | 1.0039 | .02 | * |
| CD4% | | 1.0163 | | 0.9745 | 1.0599 | .45 |  |
| CD4 | | 0.9949 | | 0.9918 | 0.9981 | .002 | ** |
| CD8% | | 1.0077 | | 1.0035 | 1.0119 | <.001 | *** |
| CD8 | | 0.8814 | | 0.8346 | 0.9308 | <.001 | *** |
| CD19% | | 0.9731 | | 0.938 | 1.0094 | .14 |  |
| C3 | 6.9297 | | | 1.9449 | 24.6905 | .003 | ** |
| Signif. codes: 0 ‘***’ 0.001 ‘**’ 0.01 ‘*’ 0.05 ‘.’ 0.1 ‘ ’ 1 | | | | | |  |  |
| Concordance= 0.8 (se = 0.034 ) | | |  |  |  |  |  |
