# Supplementary material for: Nomogram for Predicting COVID-19 Disease Progression Based on Single-Center Data: Observational Study and Model Development
Source: JMIR Med Inform. 2020 Sep 8;8(9):e19588. doi: 10.2196/19588 (PMC7485996; doi:10.2196/19588)
Supplement: Multimedia Appendix 3 [file medinform_v8i9e19588_app3.doc]

**How to use the nomogram**

**Instructions:** To use the nomogram, locate the first variable. Draw a line straight upwards to the Points axis to determine the number of points received for the variable. Repeat this process for the other five variables and sum up the points achieved for each variable. Sum of these numbers is located on the Total Points axis, and a line is drawn downwards to the non-severe probability axes to determine the 0.5-, 1-, 2-, 3-week non-severe probability.

**Example:** A 50 years old male patient was confirmed to be COVID-19 infection. The levels of CK, CD4 count, CD8 ratio, CD8 count, and C3 were 300 U/L, 400 /μL, 35%, 800 /μL, and 0.9 g/L respectively. These six variables (CK, CD4 count, CD8 ratio, CD8 count, and C3) got points of 12, 7, 58, 42, 38, and 16, respectively. A total of 173 points by summing of these six numbers showed that this patient had 80% 1-week non-severe probability and 35% 2-week non-severe probability.
