# Supplementary figures and images for: Nomogram for Predicting COVID-19 Disease Progression Based on Single-Center Data: Observational Study and Model Development
Source: JMIR Med Inform. 2020 Sep 8;8(9):e19588. doi: 10.2196/19588 (PMC7485996; doi:10.2196/19588)

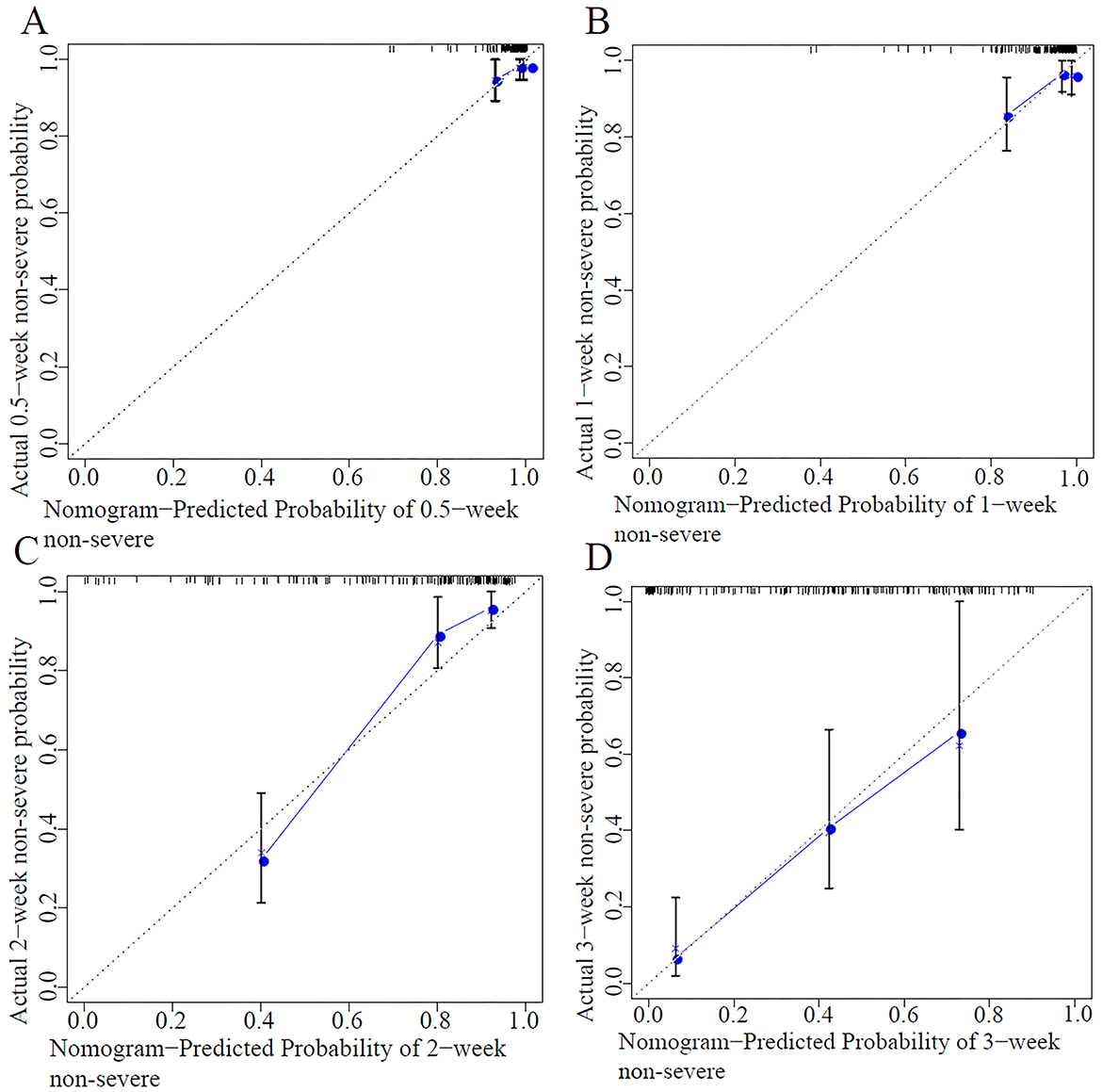

Supplement: Multimedia Appendix 4 [file medinform_v8i9e19588_app4.png]

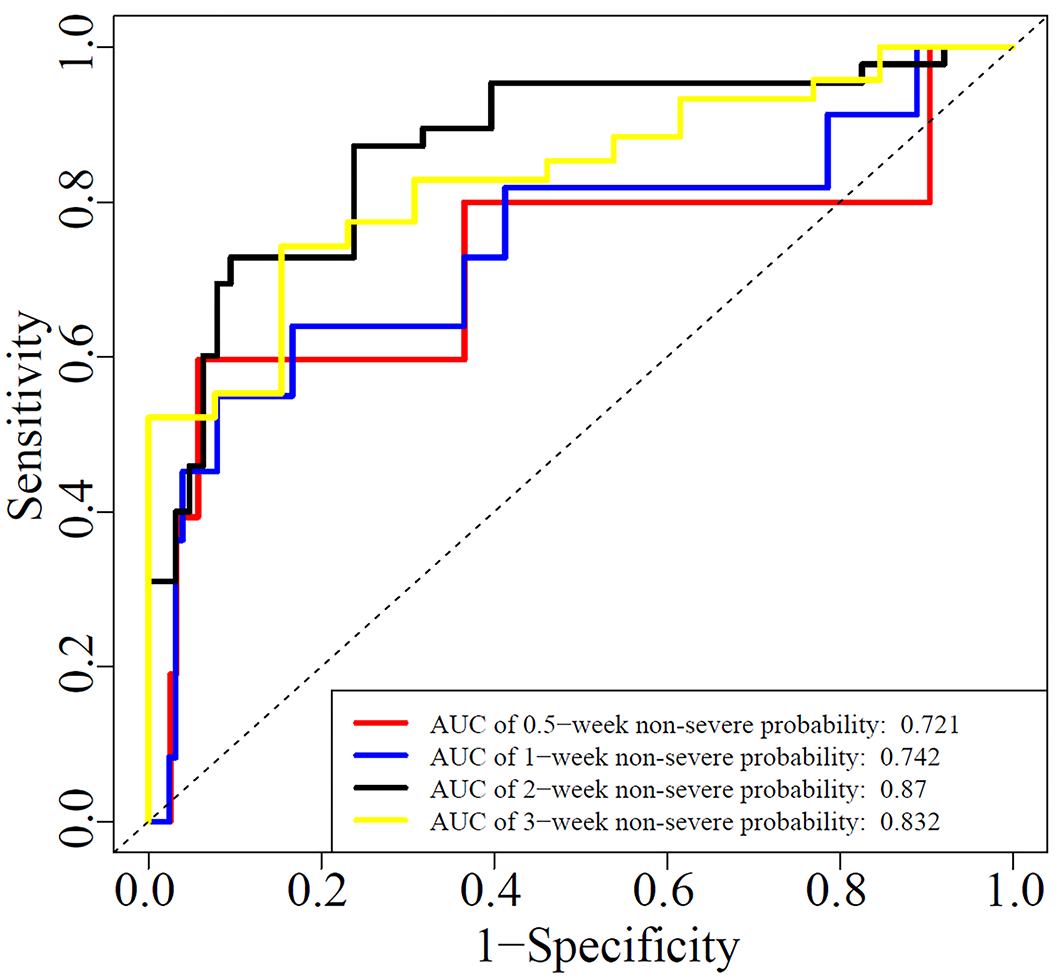

Supplement: Multimedia Appendix 5 [file medinform_v8i9e19588_app5.png]
